# Aggregated Antibiograms and Monitoring of Drug-Resistant *Streptococcus pneumoniae*

**DOI:** 10.3201/eid0909.020620

**Published:** 2003-09

**Authors:** Chris A. Van Beneden, Catherine Lexau, Wendy Baughman, Brenda Barnes, Nancy Bennett, P. Maureen Cassidy, Margaret Pass, Lisa Gelling, Nancy L. Barrett, Elizabeth R. Zell, Cynthia G. Whitney

**Affiliations:** * Centers for Disease Control and Prevention, Atlanta, Georgia, USA; † Minnesota Department of Health, Minneapolis, Minnesota, USA; ‡ Veterans Affairs Medical Center, Atlanta, Georgia, USA; § Vanderbilt Medical Center, Nashville, Tennessee, USA; ¶ Monroe County Health Department, Rochester, New York, USA; # Oregon Department of Human Services, Portland, Oregon, USA; ** Johns Hopkins Bloomberg School of Public Health, Baltimore, Maryland, USA; †† California Emerging Infections Program, Oakland, California, USA; ‡‡ Connecticut Emerging Infections Program, Department of Public Health, Hartford, Connecticut, USA

**Keywords:** *Streptococcus pneumoniae*, antibiogram, pneumococcal infections, drug resistance, population surveillance, epidemiology

## Abstract

Community-specific antimicrobial susceptibility data may help monitor trends among drug-resistant *Streptococcus pneumoniae* and guide empiric therapy. Because active, population-based surveillance for invasive pneumococcal disease is accurate but resource intensive, we compared the proportion of penicillin-nonsusceptible isolates obtained from existing antibiograms, a less expensive system, to that obtained from 1 year of active surveillance for Georgia, Tennessee, California, Minnesota, Oregon, Maryland, Connecticut, and New York. For all sites, proportions of penicillin-nonsusceptible isolates from antibiograms were within 10 percentage points (median 3.65) of those from invasive-only isolates obtained through active surveillance. Only 23% of antibiograms distinguished between isolates intermediate and resistant to penicillin; 63% and 57% included susceptibility results for erythromycin and extended-spectrum cephalosporins, respectively. Aggregating existing hospital antibiograms is a simple and relatively accurate way to estimate local prevalence of penicillin-nonsusceptible pneumococcus; however, antibiograms offer limited data on isolates with intermediate and high-level penicillin resistance and isolates resistant to other agents.

Infections from *Streptococcus pneumoniae* tax the healthcare system in the United States and other countries. Scientific advances have been made in the treatment and prevention of pneumococcal infections through antibiotics and licensure of vaccines for both adults and children; however, the last few decades have witnessed the emergence of *S. pneumoniae* resistance to antibiotics (*1*). In a multistate, population-based surveillance system that follows invasive disease from pneumococcus and other bacterial pathogens, the percent of isolates resistant to penicillin reached 24% in 1998; concurrent increases in resistance to other antimicrobial drugs have also been noted among penicillin-resistant pneumococci (*1*,*2*). Implications of drug-resistance extend beyond the laboratory and into clinical practice as treatment failures from drug resistance have been reported with meningitis (*3*–*5*) and otitis media (*6*,*7*). In some studies, increased death and disease in patients hospitalized with pneumonia caused by high-level β-lactam-resistant pneumococci have been reported (*8*,*9*).

Measuring pneumococcal resistance to penicillin and other antibiotics enables epidemiologists and healthcare providers to monitor trends, develop guidelines for optimal empiric therapy, and provide impetus for and ascertain the success of educational efforts promoting the judicious use of antibiotics. Antimicrobial resistance is not uniform across the United States (*10*). Nonsusceptibility to penicillin among invasive pneumococcal isolates has been shown to range from 15% to 35% among populations in the Centers for Disease Control and Prevention’s (CDC) national surveillance system (*1*).

The ideal method for accurate tracking of antimicrobial-resistance patterns in a community may be active, laboratory-based surveillance systems that collect strains for susceptibility testing in a reference laboratory. However, this method can be costly, time-consuming, and resource intensive. Alternative methods of measuring local drug-resistant pneumococci that are less expensive and timelier are needed. One alternative is to use aggregated antibiograms. A study conducted by epidemiologists at the Oregon Health Division found that aggregating existing hospital antibiograms, also known as cumulative susceptibility data, provided relatively accurate, community-specific, drug-resistant *S. pneumoniae* data when compared with active-, laboratory-based surveillance, the standard criterion for invasive disease. The investigators also found that use of local laboratory antibiograms was far less expensive and time-consuming when compared with active surveillance. Whether Oregon’s results can be generalized is unknown; however, only 12 hospitals in one city (Portland) were surveyed, and the percent of *S. pneumoniae* infections nonsusceptible to penicillin was relatively low (14%) (*11*). We compared the two methods of surveillance in a larger study that involved sites in geographically disparate areas and represented a larger fraction of the national population and varying degrees of drug resistance across the United States. Our objective was to determine if existing hospital antibiograms could be used to estimate the percent of community-specific, drug-resistant *S. pneumoniae* in multiple sites.

## Methods

We compared the proportions of drug-resistant *S. pneumoniae* isolates reported by participating clinical laboratories from the Active Bacterial Core Surveillance (ABCs) sites to proportions obtained by aggregation of existing antibiograms produced by the same ABCs laboratories.

### Active Laboratory-Based Surveillance

ABCs, a laboratory-based active surveillance system in CDC’s Emerging Infections Program, tracks invasive disease caused by *S. pneumoniae* and other bacterial pathogens of public health importance (*12*). Surveillance areas included in this analysis were: California (CA) (San Francisco County), Connecticut (CT) (entire state), Georgia (GA) (20-county area, including Atlanta), Maryland (MD) (6-county area including Baltimore), Minnesota (MN) (7 counties), New York (NY) (7 counties), Oregon (OR) (3-county area including Portland), and Tennessee (TN) (5 counties). The total population under surveillance was 17 million. A case of invasive pneumococcal disease was defined as the isolation of *S. pneumoniae* from a normally sterile site (e.g., blood, cerebrospinal fluid) from a surveillance area resident. Surveillance personnel routinely contacted all clinical microbiology laboratories in their site to identify cases and conducted audits every 6 months to ensure complete reporting.

Pneumococcal isolates collected through ABCs were sent to reference laboratories for susceptibility testing by broth microdilution according to the methods of the National Committee for Clinical Laboratory Standards (NCCLS) (*13*). Isolates were defined as susceptible, having intermediate resistance, or resistant to agents tested according to NCCLS definitions (*14*).

### Antibiograms

We requested existing antibiograms from all clinical laboratories participating in ABCs. The antibiograms were to cover the most recent 12-month period for which completed data were available at the time of the inquiry (1997 for GA, TN, CA, MN, OR, MD, and CT; 1998 for NY). Any identifying information (e.g., hospital name) obtained during collection of antibiogram data was removed before the data were forwarded to study investigators at CDC. Surveillance personnel also used a standardized questionnaire to query each hospital’s infection control practitioner or microbiology supervisor regarding the production and distribution of local antibiograms and whether antibiogram data included sterile isolates, nonsterile site isolates, or duplicates isolates from a single patient.

We compiled total numbers of *S. pneumoniae* isolates identified from the ABCs sites along with the percent of intermediate and resistant isolates, focusing on nonsusceptibility to penicillin, macrolides, and extended-spectrum cephalosporins (e.g., cefotaxime, ceftriaxone). We defined nonsusceptible isolates as those that were of intermediate and high-level resistance or that were simply described as not susceptible to the antibiotic tested. We aggregated data obtained from the participating hospitals within each ABCs site to produce summary antimicrobial susceptibility percentages. When generating tables comparing percent of nonsusceptible pneumococcal isolates estimated by antibiograms and by ABCs, we used only antibiogram data for the year in question (1997 for all sites excluding New York [1998]); antibiograms covering other periods were excluded from this portion of the analysis. Also, we used only antibiograms that included both the total number of isolates tested and the percent nonsusceptible for each of the antibiotics evaluated; this system allowed for aggregation of the laboratory’s data with those from other laboratories. If only a subset of isolates were tested against erythromycin and extended-spectrum cephalosporins, we excluded these results from the aggregated total for erythromycin, cephalosporins, or both. We also calculated the percent of laboratories that included *S. pneumoniae* susceptibility testing to a variety of other antimicrobial agents and the percent of laboratories generating antibiograms that included susceptibility testing of gram-negative bacteria.

To compare the proportions of resistant and susceptible *S. pneumoniae* isolates detected by the two surveillance methods, we examined the proportion of hospitals whose aggregated antibiogram data fell within a range of ± 5% and ± 10% compared with that detected through active surveillance.

## Results

### Generation of Antibiograms

One hundred and forty-five ABCs laboratories completed the surveys; these laboratories conducted antibiotic susceptibility and other testing for a total of 170 (85%) of the 199 hospital laboratories participating in ABCs at the time the study was undertaken. Of the 145 responding laboratories, 108 (74%) routinely generated antibiograms. The 108 antibiograms created include pneumococcal susceptibility testing results for 140 (70%) of the 199 ABCs hospital laboratories. In-house microbiologists typically generated the antibiograms (83%), while infection control practitioners (7%) and pharmacists (10%) created the remaining. Nearly all laboratories included both sterile site (98%) and nonsterile site (92%) isolates in the antibiograms. Ninety-five percent included inpatient, and 79% included outpatient isolates. Forty-six laboratories (43%) included duplicate isolates from individual patients in their antibiograms.

When asked how pneumococcal isolates with intermediate susceptibility were categorized, survey responders stated that their laboratory characterized these isolates as intermediate (37%), resistant (32%), susceptible (5%), and nonsusceptible (22%). This question did not specify the antibiotic tested. Only 25 (23%) laboratories generated antibiograms that included data distinguishing isolates intermediate and resistant to penicillin; 77% only indicated whether the isolates were susceptible or nonsusceptible.

The average number of isolates included in the summary antibiograms was nearly double the number collected through active surveillance; the mean number of pneumococcal isolates (per site) tested for penicillin susceptibility was 417 (range 69–850) for ABCs and 826 (range 383–1,291) for summary antibiograms. Hospitals (n=40) that excluded duplicate isolates from antibiograms averaged similar numbers of isolates (mean 89 isolates) tested for penicillin susceptibility as did hospitals (n=34) whose antibiograms included multiple isolates from a single patient (mean number of isolates tested 88).

Of the 140 hospital laboratories whose pneumococcal antibiotic susceptibility testing results were summarized in antibiograms, 96 (70%) created antibiograms with penicillin-susceptibility results in a format that could be aggregated for the year in question. The proportion of laboratories in each site that generated usable penicillin susceptibility data ranged from 70% (MD) to 100% (NY and MN). Antibiograms included susceptibility-testing results for macrolides (63%) and third-generation cephalosporins (57%). The proportion of laboratories for which this susceptibility information was in a format that could be aggregated, however, was smaller for macrolides (44%) and third-generation cephalosporins (39%). For the eight sites, the proportion of penicillin-nonsusceptible isolates from ABCs ranged from 14.5% (NY) to 38.4% (TN), whereas antibiograms yielded a range of 18.5% (CA) to 41.7% (TN) (Table 1). For all sites the overall proportion of isolates nonsusceptible to penicillin according to antibiograms was within 10 percentage points of the population- and laboratory-based surveillance (ABCs); for six sites it was within 5 percentage points. The proportion of penicillin-nonsusceptible isolates for each site identified by antibiograms was higher than that generated by ABCs (median difference: 3.65%; range 8.6% to 1.8%). No correlation existed between site-specific levels of penicillin resistance and the magnitude of difference between site-specific penicillin resistance identified by the two methods.

**Table 1:** Comparison of percent of *Streptococcus pneumoniae* isolates nonsusceptible to penicillin by site: active surveillance (ABCs) versus antibiogram

Site	Antibiogram				ABCs				
	No. laboratories^a^	Nonsusceptible isolates	Total no. isolates tested	% nonsusceptible	No. laboratories	Nonsusceptible isolates	Total no. isolates tested	% nonsusceptible	Difference in % nonsusceptible (antibiograms vs. ABCs)
Connecticut	16	168	845	19.9	32	113	624	18.1	1.8
California	9	107	577	18.5	10	30	184	16.3	2.2
Oregon	9	115	550	20.9	15	32	178	18.0	2.9
Tennessee	10	432	1,037	41.7	31	169	440	38.4	3.3
Maryland	10	171	886	19.3	27	85	557	15.3	4.0
Georgia	14	505	1,291	39.1	39	292	850	34.4	4.7
New York	9	85	383	22.2	20	10	69	14.5	7.7
Minnesota	19	315	1,037	30.4	25	95	435	21.8	8.6
Total	96	1,898	6,606	28.7	199	826	3,337	24.8	Median: 3.65

^a^Only laboratories whose antibiograms covered the calendar year in question (1997 for all sites except NY [1998]) were compared to ABCs.

The proportions of pneumococcal isolates nonsusceptible to a third-generation cephalosporin and to erythromycin were lower than the proportion of penicillin-nonsusceptible isolates, regardless of the method used (Tables 2 and 3). Similar to the results for penicillin, the percent of strains nonsusceptible to third-generation cephalosporins or erythromycin as detected by antibiograms tended to be greater than the percent nonsusceptible detected by ABCs. In contrast, the range of the differences for third-generation cephalosporins and erythromycin detected by the two surveillance methods was larger than the range of differences for penicillin as measured for each ABCs site. The magnitude of the difference in overall susceptibility to third-generation cephalosporins determined by the two surveillance methods was <10% for seven of eight sites and <5% for five sites. The magnitude of the difference in susceptibility to erythromycin as determined by the two surveillance methods was <10% for all sites and <5% for only four sites.

**Table 2:** Comparison of percent of *Streptococcus pneumoniae* isolates nonsusceptible to third-generation cephalosporins by site: active surveillance (ABCs) versus antibiograms

Site	Antibiogram				ABCs				Difference in % nonsusceptible (antibiograms vs. ABCs)
	No. laboratories^a^	Non-susceptible isolates	Total no. isolates tested	% non-susceptible	No. laboratories	Non-susceptible isolates	Total no. isolates tested	% non-susceptible	
Tennessee	10	54	357	15.1	31	114	440	25.9	–10.8
New York	3	2	84	2.4	20	5	69	7.2	–4.8
California	4	14	412	3.4	10	15	184	8.1	–4.7
Connecticut	5	19	267	7.1	32	73	624	11.7	–4.6
Oregon	6	34	419	8.1	15	14	178	7.9	0.2
Maryland	5	53	476	11.1	27	48	557	8.6	2.5
Minnesota	7	104	543	19.2	25	60	435	13.8	5.4
Georgia	14	222	1272	17.5	39	102	850	12.0	5.5
Total	54	502	3,830	13.1	199	431	3,337	12.9	Median: –2.25

^a^Only laboratories whose antibiograms covered the calendar year in question (1997 for all sites except NY [1998]) were compared to ABCs.

**Table 3:** Comparison of percent of *Streptococcus pneumoniae* isolates nonsusceptible to erythromycin by site: active surveillance (ABCs) versus antibiogram

Site	Antibiogram				ABCs				Difference in % nonsusceptible (antibiogram vs. ABCs)
	No. laboratories ^a^	Non -susceptible isolates	Total. isolates tested	% non-susceptible	No. laboratories	Non-susceptible isolates	Total isolates tested	% non-susceptible	
Georgia	10	178	805	22.1	39	207	850	24.4	-2.3
Tennessee	8	133	460	28.9	31	113	440	25.7	3.2
Oregon	6	57	405	14.1	15	18	178	10.1	4.0
Maryland	7	64	596	10.7	27	35	557	6.3	4.4
New York	4	23	128	11.7	20	4	69	5.8	5.9
California	9	92	577	15.9	10	15	184	8.2	7.7
Minnesota	10	140	684	20.5	25	55	435	12.6	7.9
Connecticut	7	58	287	20.2	32	65	624	10.4	9.8
Total	61	737	3,942	18.7	199	512	3,337	15.3	Median: 5.15

^a^Only laboratories whose antibiograms covered the calendar year in question (1997 for all sites except NY [1998]) were compared to ABCs.

In addition to penicillin, cephalosporins, and macrolides, submitted antibiograms included susceptibility testing results for a variety of other antibiotics that included the following: trimethoprim/sulfamethoxazole (35%), vancomycin (59%), clindamycin (47%), gentamycin (3.9%), and one or more fluoroquinolones (14%). Thirty-eight percent of antibiograms returned for analysis also included antimicrobial susceptibility testing results for various gram-negative bacteria.

## Discussion

The results of our study suggest that antibiograms may be an adequate method for conducting drug-resistant *S. pneumoniae* surveillance for many health departments, illustrating the comparability of aggregated antibiograms that include both sterile and nonsterile site isolates to active, laboratory- and population-based surveillance for invasive isolates. For more than half the comparisons between the two methods, the difference in antibiotic resistance detected was <5 percentage points, and for 23 (96%) of the 24 comparisons the difference was <10 percentage points. No significant differences in comparability of the two methods were noted between high- and low-resistance areas. This study indicates that antibiograms may be an alternative tool for evaluating penicillin nonsusceptibility in a region and validates the earlier findings of the Oregon study, conducted in an area of relatively low antibiotic resistance (*11*).

Although the estimates of level of resistance obtained from antibiograms approximated that from ABCs, aggregated antibiogram data tended to show a higher proportion of nonsusceptible isolates within each site and for each antibiotic evaluated. This trend is likely due to the inclusion of nonsterile (noninvasive) site isolates. In studies from centers that include both sterile and nonsterile isolates, nonsterile site isolates have been found to be equally or more resistant (*15*–*17*). The reason for this difference is unclear but may reflect differences in serotype distribution between strains causing invasive and noninvasive disease. Disparity in results between clinical and reference laboratories could also contribute to this trend; use of the E test (AB Diodisk, Solna, Sweden) by clinical laboratories might vary from the referent method (broth microdilution) by one half or one dilution (*18*). In this study, we were unable to examine the role of laboratory error or differences in susceptibility-testing methods as a reason for differences in results from antibiograms compared with those from active surveillance.

Compared to penicillin, differences between the two surveillance methods were greater for extended-spectrum cephalosporins and erythromycin. This finding may be because of smaller numbers of isolates included in the antibiograms, fewer laboratories that included susceptibility testing of *S. pneumoniae* to these antibiotics, or greater disagreement between clinical and reference laboratory results. We could not include antibiogram-susceptibility testing results for some hospital laboratories because only a subset of the pneumococcal isolates that were tested for penicillin nonsusceptibility were also tested for susceptibility against third-generation cephalosporins (20 laboratories) and erythromycin (13 laboratories). The potential explanations for why these laboratories tested only a subset of pneumococcal isolates (i.e., only penicillin-nonsusceptible isolates were tested) against the same antibiotics were not indicated on the antibiograms.

We chose to evaluate the comparability of the two surveillance methods by observing how often the percent nonsusceptible isolates estimated by aggregated antibiograms differed by <5 and 10 percentage points from that estimated by ABCs active surveillance. As there exists no standardized or absolute level of antimicrobial drug resistance that would dictate a change in empiric treatment of pneumococcal infections, we chose a priori two conservative thresholds of difference. A healthcare provider may not modify empiric therapy based on the differences found in our study, and the magnitude of differences reported here are likely not relevant from a public health perspective. Trends of pneumococcal antibiotic resistance over time may be of more clinical and epidemiologic relevance than an absolute level. Knowledge of local trends may help communities assess regional antibiotic use and evaluate the effects of local educational measures promoting the judicious use of antibiotics. As this study did not span multiple years, we could not document the ability of antibiograms to detect trends. However, given that sentinel surveillance conducted in ABCs sites has been shown to detect pneumococcal resistance trends over time (*19*) and that in our study antibiograms provided site-specific point estimates of antibiotic resistance similar to those measured by active sruveillance, antibiograms may be able to follow trends in pneumococcal antimicrobial resistance at the local level.

Drawbacks to this surveillance method include the inability to evaluate resistance to multiple drugs. Relatively few drugs can be evaluated because of laboratory variations in antibiotics selected for susceptibility testing by antibiograms. Health departments that wish to monitor emerging resistance patterns to antibiotics, such as vancomycin or fluoroquinolones, might consider a method other than aggregated susceptibility tables, or they might encourage hospital laboratories within a defined community to standardize their susceptibility panels to facilitate aggregation of results. Another limitation of antibiograms is the inability to distinguish between intermediate- and high-level resistance to penicillin; 77% of antibiograms in our study expressed resistance as percent nonsusceptible rather than distinguishing between intermediate and resistant isolates. This distinction has become relevant for treatment of some infections. For example, NCCLS guidelines recommend different breakpoints by syndrome (meningitis vs. nonmeningitis) for some agents (*20*). Aggregating antibiograms is useful for infections that are generally community-acquired, but antimicrobial resistance in hospital-acquired infections should be evaluated based on the knowledge of the particular institution’s flora. Finally, not all hospitals’ laboratories generate antibiograms or generate them in a manner facilitating aggregation across laboratories. However, we demonstrated the comparability of the two surveillance methods despite the fact that the penicillin-nonsusceptibility results, as measured by antibiograms, was known for only 96 (48%) of the 199 ABCs hospital laboratories.

This study should help clinicians and public health personnel in state or local health departments determine which surveillance tool for obtaining estimates of antibiotic-resistant *S. pneumoniae* is best suited to their specific region or community by providing background information on two alternative systems; the benefits and limitations of each system may be reviewed to determine the most useful and practical surveillance tool for a particular region. Antibiograms are relatively inexpensive and easy to use. Although not measured in our study, epidemiologists in Oregon found that the cost of active surveillance was approximately 70 times that of aggregating antibiograms for the three-county study area (*11*); the high cost of this type of surveillance, however, is partially due to the fact that ABCs is an integrated system that accomplishes multiple objectives in addition to susceptibility testing of pneumococcal isolates (*12*). Most hospitals and laboratories routinely generate antibiograms; therefore, obtaining this information is relatively easy and within the capacity of local health departments. Active surveillance, on the other hand, excludes duplicate isolates for a single patient or infection and is able to provide extensive additional information such as risk factors for resistant infections, outcome data, and other laboratory testing such as serotype determination. Active surveillance also limits case and isolate collection to persons who are residents of the defined surveillance area, allowing for calculation of rates of disease. Furthermore, active surveillance provides individual patient-level data, allowing assessment of the impact of specific interventions such as pneumococcal conjugate vaccination of infants and young children. Attainment of patient-level data through active surveillance also permits detection of possible changes in the incidence of resistant pneumococcal infections (e.g., because of a general decrease in cases of pneumococcal infection among children receiving pneumococcal conjugate vaccine) that might go unnoticed if only the proportion of resistant isolates were tracked (i.e., as done by antibiograms).

Increasing antibiotic drug resistance is a problem that is global in scale and that has practical implications for the treatment and outcome of invasive infections from *S. pneumoniae* and other bacteria of public health importance. Clinicians and researchers are now acknowledging the importance of preventing resistant infections through appropriate use of antibiotics and vaccines. Surveillance data are needed to monitor the success of these campaigns and to raise awareness of the problem. Because most local laboratories generate antibiograms routinely, collecting aggregating antibiogram data is an inexpensive and readily available method of measuring local antibiotic resistance levels. Although providing less information than active surveillance, aggregated antibiogram data are a generally accurate way for health departments to generate needed community-specific estimates of pneumococcal resistance.

## References

[1] Whitney CG, Farley MM, Hadler J, Harrison LH, Lexau C, Reingold A, Increasing prevalence of multidrug-resistant *Streptococcus pneumoniae* in the United States. N Engl J Med. 2000;343:1917–24. 10.1056/NEJM200012283432603 11136262

[2] Kaplan SL, Mason EO Jr, Wald E, Tan TQ, Schutze GE, Bradley JS, Six year multicenter surveillance of invasive pneumococcal infections in children. Pediatr Infect Dis J. 2002;21:141–7. 10.1097/00006454-200202000-00011 11840082

[3] Kaplan SL, Mason EO Jr. Management of infections due to antibiotic-resistant *Streptococcus pneumoniae.* Clin Microbiol Rev. 1998;11:628–44. 9767060 10.1128/cmr.11.4.628 PMC88901

[4] Heffelfinger JD, Dowell SF, Jorgensen JH, Klugman KP, Mabry LR, Musher DM, Management of community-acquired pneumonia in the era of pneumococcal resistance: a report from the Drug-Resistant *Streptococcus pneumoniae* Therapeutic Working Group. Arch Intern Med. 2000;160:1399–408. 10.1001/archinte.160.10.1399 10826451

[5] John CC. Treatment failure with use of a third-generation cephalosporin for penicillin-resistant pneumococcal meningitis: case report and review. Clin Infect Dis. 1994;18:188–93. 8161625 10.1093/clinids/18.2.188

[6] Dagan R, Abramson O, Leibovitz E, Lang R, Goshen S, Greenberg D, Impaired bacteriologic response to oral cephalosporins in acute otitis media caused by pneumococci with intermediate resistance to penicillin. Pediatr Infect Dis J. 1996;15:980–5. 10.1097/00006454-199611000-00010 8933545

[7] del Castillo F, Baquero-Artigao F, Garcia-Perea A. Influence of recent antibiotic therapy on antimicrobial resistance of Streptococcus pneumoniae in children with acute otitis media in Spain. Pediatr Infect Dis J. 1998;17:94–7. 10.1097/00006454-199802000-00003 9493802

[8] Feikin DR, Schuchat A, Kolczak M, Barrett NL, Harrison LH, Lefkowitz L, Mortality from invasive pneumococcal pneumonia in the era of antibiotic resistance, 1995–1997. Am J Public Health. 2000;90:223–9. 10.2105/AJPH.90.2.223 10667183 PMC1446155

[9] Turett GS, Blum S, Fazal BA, Justman JE, Telzak EE. Penicillin resistance and other predictors of mortality in pneumococcal bacteremia in a population with high human immunodeficiency virus seroprevalence. Clin Infect Dis. 1999;29:321–7. 10.1086/520209 10476736

[10] Thornsberry C, Sahm DF, Kelly LJ, Critchley IA, Jones ME, Evangelista AT, Regional trends in antimicrobial resistance among clinical isolates of *Streptococcus pneumoniae, Haemophilus influenzae*, and *Moraxella catarrhalis* in the United States: results from the TRUST Surveillance Program, 1999–2000. Clin Infect Dis. 2002;34(Suppl 1):S4–16. 10.1086/324525 11810606

[11] Chin AE, Hedberg K, Cieslak PR, Cassidy M, Stefonek KR, Fleming DW. Tracking drug-resistant *Streptococcus pneumoniae* in Oregon: an alternative surveillance method. Emerg Infect Dis. 1999;5:688–93. 10.3201/eid0505.990510 10511525 PMC2627721

[12] Schuchat A, Hilger T, Zell E, Farley MM, Reingold A, Harrison L, Active Bacterial Core Surveillance of the Emerging Infections Program Network. Emerg Infect Dis. 2001;7:92–9. 10.3201/eid0701.010114 11266299 PMC2631675

[13] National Committee for Clinical Laboratory Standards. Approved standard M7-A4: standard methods for dilution antimicrobial susceptibility tests for bacteria that grow aerobically. Wayne (PA): The Committee; 1997.

[14] National Committee for Clinical Laboratory Standards. Performance standards for antimicrobial susceptibility testing: M100-S8. Wayne (PA): The Committee; 1998.

[15] Doern GV, Heilmann KP, Huynh HK, Rhomberg PR, Coffman SL, Brueggemann AB. Antimicrobial resistance among clinical isolates of *Streptococcus pneumoniae* in the United States during 1999–2000, including a comparison of resistance rates since 1994–1995. Antimicrob Agents Chemother. 2001;45:1721–9. 10.1128/AAC.45.6.1721-1729.2001 11353617 PMC90537

[16] Doern GV, Brueggemann AB, Huynh HK, Wingert E, Rhomberg PR. Antimicrobial resistance with *Streptococcus pneumoniae* in the United States, 1997–1998. Emerg Infect Dis. 1999;5:757–65. 10.3201/eid0506.990603 10603208 PMC2640814

[17] Heffernan R, Henning K, Labowitz A, Hjelte A, Layton M. Laboratory survey of drug-resistant *Streptococcus pneumoniae* in New York City, 1993–1995. Emerg Infect Dis. 1998;4:113–6. 10.3201/eid0401.980116 9452405 PMC2627676

[18] Tenover FC, Baker CN, Swenson JM. Evaluation of commercial methods for determining antimicrobial susceptibility of *Streptococcus pneumoniae.* J Clin Microbiol. 1996;34:10–4. 8748262 10.1128/jcm.34.1.10-14.1996 PMC228719

[19] Schrag SJ, Zell ER, Schuchat A, Whitney CG. Sentinel surveillance: a reliable way to track antibiotic resistance in communities? Emerg Infect Dis. 2002;8:496–502. 11996685 10.3201/eid0805.010268 PMC2732493

[20] National Committee for Clinical Laboratory Standards. Performance standards for antimicrobial susceptibility testing; twelfth informational supplement. NCCLS document M100-S12 (ISBN 1-56238-454-6). Wayne (PA): The Committee; 2002.

